# Tobacco Smoke Induces and Alters Immune Responses in the Lung Triggering Inflammation, Allergy, Asthma and Other Lung Diseases: A Mechanistic Review

**DOI:** 10.3390/ijerph15051033

**Published:** 2018-05-21

**Authors:** Agnieszka Strzelak, Aleksandra Ratajczak, Aleksander Adamiec, Wojciech Feleszko

**Affiliations:** Department of Pediatric Pulmonology and Allergy, Medical University of Warsaw, Zwirki i Wigury 61, 02-091 Warszawa, Poland; agnieszka@strzelak.pl (A.S.); olarurarz@gmail.com (A.R.); alekadamiec@gmail.com (A.A.)

**Keywords:** tobacco, cigarette, smoke, lung, airway, immunity, asthma, atopy, allergy, children

## Abstract

Many studies have been undertaken to reveal how tobacco smoke skews immune responses contributing to the development of chronic obstructive pulmonary disease (COPD) and other lung diseases. Recently, environmental tobacco smoke (ETS) has been linked with asthma and allergic diseases in children. This review presents the most actual knowledge on exact molecular mechanisms responsible for the skewed inflammatory profile that aggravates inflammation, promotes infections, induces tissue damage, and may promote the development of allergy in individuals exposed to ETS. We demonstrate how the imbalance between oxidants and antioxidants resulting from exposure to tobacco smoke leads to oxidative stress, increased mucosal inflammation, and increased expression of inflammatory cytokines (such as interleukin (IL)-8, IL-6 and tumor necrosis factor α ([TNF]-α). Direct cellular effects of ETS on epithelial cells results in increased permeability, mucus overproduction, impaired mucociliary clearance, increased release of proinflammatory cytokines and chemokines, enhanced recruitment of macrophages and neutrophils and disturbed lymphocyte balance towards Th2. The plethora of presented phenomena fully justifies a restrictive policy aiming at limiting the domestic and public exposure to ETS.

## 1. Introduction

Despite many efforts to reduce its prevalence, approximately six million people worldwide die due to tobacco use each year [1]. About 600,000 of them die from the effects of second-hand smoke. Apart from contributing to the pathogenesis of chronic obstructive pulmonary disease (COPD), hypertension, cardiovascular disease and cancer, cigarette smoking is a recognized risk factor for many chronic systemic diseases with inflammatory components such as atherosclerosis, Crohn’s disease, rheumatoid arthritis, psoriasis, Graves’ ophthalmopathy, and noninsulin-dependent diabetes mellitus [2,3]. Furthermore, smokers show increased susceptibility towards microbial infections (respiratory tract infections [RTI], bacterial meningitis and periodontitis) and poorer wound healing [2,3,4]. For these reasons, the capacity of cigarette smoke (CS) to distort immune homeostasis has gained much attention recently.

In particular, great steps have been taken in the recent years to understand how cigarette smoke induces changes in the immune cell function in COPD patients. However, cigarette smoke induced alternations in immune responses in children, the most vulnerable population, remain an understudied area of focus. Current estimates are that 40–50% of all children across the world are exposed to second-hand smoke [5,6]. While cigarette smoke exposure (CSE) has been linked with an increased prevalence of childhood allergic diseases, the exact molecular mechanisms behind a skewed inflammatory profile that promote the development of allergy in smoke-exposed children are not completely understood. This review will focus on the effects of cigarette smoke on innate and adaptive immunity that can contribute to the development and exacerbation of allergic diseases in children. Due to the relative paucity of research in this area, the majority of reviewed mechanisms are based on the results of smoker and/or COPD patient studies and experimental models.

## 2. Exposure to Cigarette Smoke and Allergy

**Figure 1:**
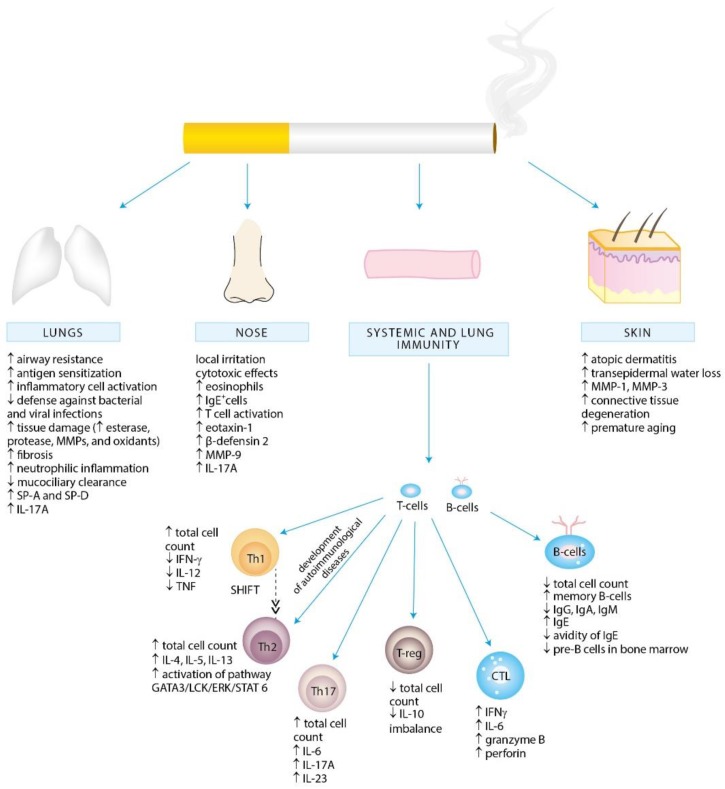
Cigarette smoke exposure-associated alternations in local and systemic immunity promoting inflammation and allergy development.

Several separate lines of evidence suggest a positive association between cigarette smoke exposure and the development of allergy (Figure 1). In particular, exposure to parental smoking, both pre- and post-natally, can contribute to allergic disorders later in life. Second-hand smoke (SHS) is a well-known contributor to respiratory tract infections, otitis media, sudden infant death syndrome, as well as behavioral and cognitive problems in children [7,8]. SHS exposure is associated with increased incidence and severity of respiratory tract infections, such as respiratory syncytial virus (RSV) infection in neonates or pneumonia in younger children [9]. While early RSV infection is an important risk factor for recurrent wheezing or childhood asthma, SHS is estimated to contribute to 165,000 annual deaths from pneumonia in children under the age of 5 years [6,10].

Mechanistically, it appears plausible that exposure to tobacco smoke can skew immune responses by impairing Th1-type and augmenting Th2-dependent responses, mainly by altering the immune functions of a variety of immune cells and aggravating the allergic inflammation and sensitization [3,11,12,13,14,15,16,17,18,19]. In line with this paradigm, Bozinovski et al. has recently demonstrated that cigarette smoke exposure promotes the release of interleukin (IL)-17A, a proinflammatory cytokine implicated in the pathogenesis of asthma, from nonconventional T-cell sources, such as natural killer (NK), natural killer T-cells (NKT) and γδ T-cells [20].

A vast body of evidence supports the linkage between parental smoking and markers of atopy in children, such as serum IgE level, eosinophilia, and positive skin-prick tests [21,22,23]. Of note, adult active smokers were also shown to display elevated serum IgE level [24]. Although systematic review from 1998 by Strachan and Cook on relationship between ETS exposure and allergic sensitization did not provide conclusive evidence, our more recent report demonstrated a positive correlation between exposure to cigarette smoke and increased levels of IgE concentrations and positive skin prick sensitization in children [25,26]. Recently, cigarette smoke exposure was significantly associated with IgE sensitization to cockroaches, grass pollen, and certain food allergens in children, and dose-dependent relationships were suggested [27]. Furthermore, children of smoking parents have been shown to have increased risk of wheeze, asthma, airways hyperresponsiveness, and allergic rhinitis [28,29,30,31,32].

In particular, epidemiological data link smoking with the development and the severity of asthma [33,34]. Active smoking worsens symptoms of asthma, precipitates decline in lung function and impairs therapeutic response to corticosteroids [33,35,36,37]. Of note, early changes in the airways were observed already in active smokers among adolescents with a short smoking history [38]. Concurrently, ETS exposure early in life is related to reduced lung function, asthma in childhood, and adult-onset asthma [39]. According to Burke et al., passive smoking increases the incidence of wheeze and asthma in children by at least 20% [40]. Further to this, exposure to tobacco smoke in early childhood impairs lung development, thus establishing an increased lifelong risk of poor lung health [41,42]. As in case of other smoke-induced diseases, however, an individual susceptibility to develop impaired lung function as a consequence of ETS exposure seems to have a non-negligible impact. Indeed, recently published results of the first genome-wide gene-by-ETS interaction study underlie three pathways through which exposure to cigarette smoke may potentially contribute to asthma development, namely the apoptosis, p38, mitogen-activated protein kinase (MAPK) and TNF pathways. All of them have been already implicated in impaired lung function in COPD [43].

Much ambiguity remains surrounding the association of cigarette smoke exposure and allergic rhinitis. While passive cigarette smoke exposure was associated with increased nasal congestion and current symptoms of rhinitis and rhinoconjunctivitis in children, other studies yielded counter results [44,45,46,47]. In adults, smoking worsens the symptoms of allergic rhinitis and increases the incidence of nasal polyposis in patients with perennial allergic rhinitis [48]. Cigarette smoke exerts cytotoxic and both proinflammatory and anti-inflammatory effects on nasal epithelial cells leading to increased reactive oxygen species (ROS) production, Toll-like receptor (TLR) 4 expression, lipopolysaccharide (LPS) and IL-17A synthesis [49,50,51]. Subsequent changes in sinonasal composition of immune cells include increased counts of neutrophils and monocyte-derived dendritic cells (DCs) demonstrated in nasal epithelium exposed to CS as well as increased eotaxin-1 immunoreactive cells and eosinophils observed in children and adolescents with perennial allergic rhinitis [50,52,53]. Priming of monocyte-derived DCs may further contribute to the development of allergic disease, since this subset of DCs has been shown to play a critical role in driving Th2 inflammation [54,55]. Furthermore, cigarette smoke exposure increases a host susceptibility to pathogen infection through disrupting the secretion of antimicrobial peptides already in nasal epithelium. Primary human nasal epithelial cells exposed to cigarette smoke secreted less chemokine ligand (C-C motif) 20, SLPI, and β-defensin 1 [56]. Importantly, cigarette smoke exposure has been recently associated with blunted nasal host defense against *Streptococcus pneumonia*, which facilitated invasive pneumococcal disease [57].

Active and passive smoking are significantly associated with atopic dermatitis in children and adults [58,59]. Moreover, SHS exposure in childhood has been shown to be associated with the development of adult-onset atopic dermatitis [60]. A variety of chemicals present in cigarette smoke enhances transepidermal water loss, upregulation of matrix metalloproteases (MMP) MMP-1 and MMP-3 implicated in collagen and elastic fibers degradation which, together with induced oxidative stress, contribute to the degeneration of connective tissue and premature aging in the skin [61]. Benzopyrene, a major polyaromatic hydrocarbon constituent of cigarette smoke, has been recently shown to regulate Langerhans cell migration and Th2- and Th17-profile cytokine production during epicutaneous sensitization response [62]. This Th2/Th17 polarization emphasizes the capacity of cigarette smoke to promote the development of atopic dermatitis. Furthermore, cigarette smoke is strongly associated with contact dermatitis [63]. It is attributable to the metal constituents of smoke fume, which act as an adjuvant in immune response to allergens and stimulate sensitization [64].

### Prenatal Exposure to Cigarette Smoke and Allergy

**Figure 2:**
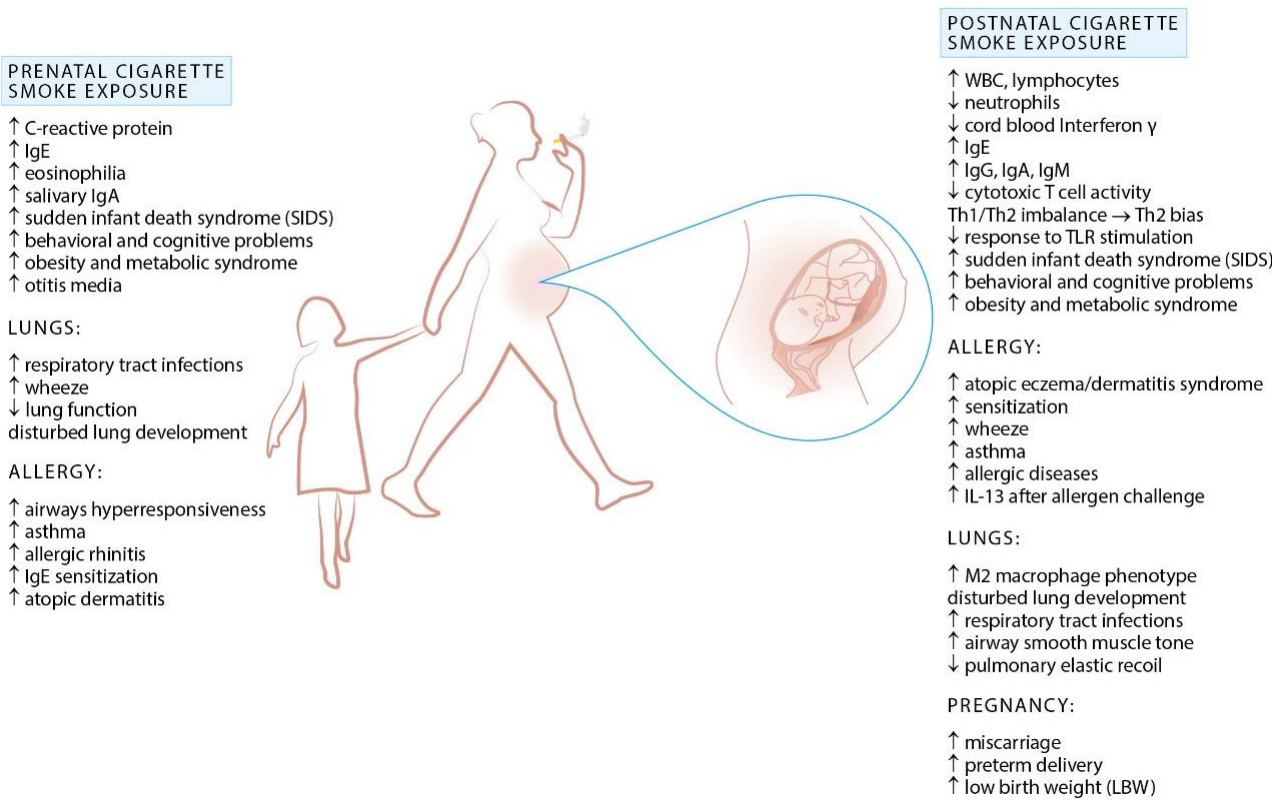
Effects of cigarette smoke exposure during prenatal life and early childhood.

In utero tobacco exposure remains common, occurring in circa 1 in 10 pregnancies [65]. A number of studies associates prenatal (maternal) smoking and ETS exposure in early childhood with decreased lung function, increased susceptibility to upper and lower respiratory tract infections (common cold, otitis media, bronchitis, pneumonia), sudden infant death syndrome, wheeze and asthma (Figure 2) [4,66,67,68,69,70]. While maternal smoking in pregnancy increases the risk of wheeze and asthma, according to recent meta-analyses it has no effect on the risk of allergic rhinitis, atopic dermatitis, and food allergy in the offspring [71].

However, prenatal nicotine exposure was reported to alter normal lung development through upregulation of nicotinic acetylcholine receptors (nAChRs) expressed in the lung and brain during early fetal life [42]. In particular, increased expression of a7 receptors in airway bronchial, cartilage and endothelial cells of fetal lung may potentially lead to enhanced differentiation of embryonic stem cells into fibroblasts, impaired lung growth and alveolar development associated with decreased lung function and lung hypoplasia in the offspring. This may, at least in part, explain why exposed neonates are at increased risk for reduced lung function, altered central and peripheral respiratory chemoreception, and increased asthma symptoms throughout childhood.

Additionally, maternal smoking during pregnancy affects immune responses in the offspring. Altered cytokine profile is detectable already in cord blood of neonates of smoking mothers, with decreased IFN-γ and increased IgE and IL-13 levels underpinning the capacity of prenatal cigarette smoke exposure to increase the risk of infections and to potentially induce a proclivity for allergy in early life [72,73]. Furthermore, neonates of smoking mothers appear to display an altered immune cells profile. Hinz et al. has proven that passive smoking during pregnancy contributes to reduced Treg-cell numbers in cord blood, which may result in higher prevalence of neonatal atopic dermatitis and food allergy [74]. Alternations in lymphocyte subsets in response to cigarette smoke exposure in utero remains, however, a matter of debate. While maternal smoking was shown to be associated with stronger neonatal lymphoproliferation, no significant differences in lymphocyte subpopulations between newborns of smoking and nonsmoking mothers were observed [75,76]. Nevertheless, in utero cigarette smoke exposure was correlated with impaired Th1 responses to polyclonal stimulation and augmented Th2 differentiation along with enhanced Th2-type cytokine production, a known contributors to allergic inflammation [73,77]. In animal studies prenatal secondhand cigarette smoke exposure increased allergen-induced airway resistance, activated the Th2-polarizing pathway, impaired mucociliary clearance and blunted Th1 responses in the offspring [78]. This has implications for allergic risk, as impaired Th1 function in the perinatal period has been associated with allergic risk in many studies [79,80]. Additionally, Th1/Th2 imbalance may account for the observed increased risk of respiratory infections in children of smoking mothers. Consistent with these observations are findings by Noakes et al., who demonstrated attenuated cytokine responses following TLRs activation in infants of smoking mothers [81]. Since TLR activation is crucial in Treg cells activation, which account for the suppression of Th2 immune responses, blunted TLR-mediated responses could further contribute to increased allergic risk.

Although both prenatal and early postnatal exposures to cigarette smoke are recognized risk factors for atopy and respiratory infections early in life, in utero exposure appears to carry a higher risk. In a study conducted on allergic asthmatic mice it has been demonstrated that prenatal but not postnatal exposure to tobacco smoke exacerbates airway reactivity, strongly upregulates allergen-induced Th2 cytokine expression, and increases total serum IgE levels [78]. Similarly, an increased risk of wheezing among children within the Generation R cohort was sustained for longer time in those exposed prenatally [82].

Once again, debilitating effects of prenatal tobacco smoke exposure are derivatives of exposure, individual genetic susceptibility, and altered epigenetic mechanisms including histone acetylation, expression of microRNA, and DNA methylation. Children with the glutathione S-transferase GSTM1-null genotype born to smoking mothers have an increased risk of early-onset and persistent asthma compared with children with protective GST genotype or those not exposed to tobacco smoke during fetal life [83,84]. Intriguingly, smoke-related epigenome modifications appear to have longitudinal consequences, as exemplified by grandmaternal effect on asthma risk observed in Children’s Health Study in southern California [31]. Although this American case-control study has demonstrated that grandmaternal smoking during the mother’s fetal period was associated with increased asthma risk in grandchildren, more recent data from Avon Longitudinal Study of Parents and Children failed to support this hypothesis. However, some evidence of an increase in asthma risk with paternal prenatal exposure was noticed [85]. A better understanding of transgenerational changes in lung development is warranted given its remote consequences.

To sum up, the relationship between cigarette smoking and asthma and other allergic disorders is complex and still not completely elucidated. Therefore tobacco smoke-induced allergic sensitization continues to be the subject of many investigations. Hopefully, tobacco control policies will have a capacity to improve the well-being of children with asthma, and potentially other allergic diseases [86].

## 3. Molecular Aspects of Tobacco Smoke Toxicity

Cigarette smoke consists of more than 7000 different chemical compounds, most of which exert adverse effects on the cells of respiratory tract. Apart from over 50 known human carcinogens (methylcholanthrene, benzo-α-pyrenes, acrolein) tobacco smoke contains toxins (carbon monoxide, ammonia, acetone, nicotine, hydroquinone), chemically reactive solids, and oxidants (superoxide, hydrogen peroxide, nitrogen oxides) [87,88].

Tobacco smoke compounds can directly influence functioning of the lung cells, demonstrating pro-inflammatory, cytotoxic, mutagenic and carcinogenic properties. In particular, inhalation of oxidants results in direct lung damage and activation of inflammatory responses leading to further tissue injury. Oxidative stress plays a pivotal role in the pathogenesis of many inflammatory lung disorders such as asthma, COPD, idiopathic pulmonary fibrosis (IPF), cystic fibrosis (CF) and adult respiratory distress syndrome (ARDS) [89,90,91,92]. An increased oxidative burden in smokers results from both reactive oxygen and reactive nitrogen species (RNS) derived from inhaled tobacco smoke and released into the milieu of the lungs by activated inflammatory cells—macrophages, epithelial cells, neutrophils, and T lymphocytes. The interaction between the cigarette smoke and the epithelial lining fluid (ELF) enables further production of ROS in the airways, since both cigarette smoke and ELF contain metal ions, such as iron, which catalyze the production of free radicals [93].

The imbalance between oxidants and antioxidants in favor of the former results in direct damage to lipids, proteins, nucleic acids and components of the lung matrix (e.g., elastin and collagen). Other consequences of oxidative stress include increased apoptosis, impairment of skeletal muscle function, mucus hypersecretion, and decreased binding affinity and translocation of steroid receptors [93]. Increased levels of ROS contribute to inactivation of antiproteases (such as a1-antitrypsin) and activation of metalloproteases (MMPs), resulting in protease/antiprotease imbalance in the lungs, which directly contributes to the degradation of the lung matrix [94]. Additionally, cigarette smoking depletes the level of glutathione (GSH), a major antioxidant of the lung [95]. Changes in the redox status within the cell initiate the lung inflammatory responses through enhancement of the respiratory burst in phagocytic cells, regulation of intracellular signalling, chromatin remodeling (histone acetylation/deacetylation) and activation of redox-sensitive transcription factors, such as nuclear factor-κB (NF-κB) and activator protein-1 (AP-1). The latter are critical to gene expression of pro-inflammatory mediators such as interleukin (IL)-8, IL-6, and tumor necrosis factor-α (TNF-α) which links cigarette smoke exposure with altered cytokine production [90].

Other pathophysiological mechanisms by which cigarette smoke can alter cytokine gene transcription rely on smoke-induced changes to the epigenome, such as DNA methylation, expression of microRNA and histone modification [96,97]. CSE-associated epigenetic modifications underlying allergic and respiratory diseases are, however, beyond the scope of this review and are discussed in detail elsewhere [98].

## 4. Effects of Cigarette Smoke Exposure on the Immune System

**Figure 3:**
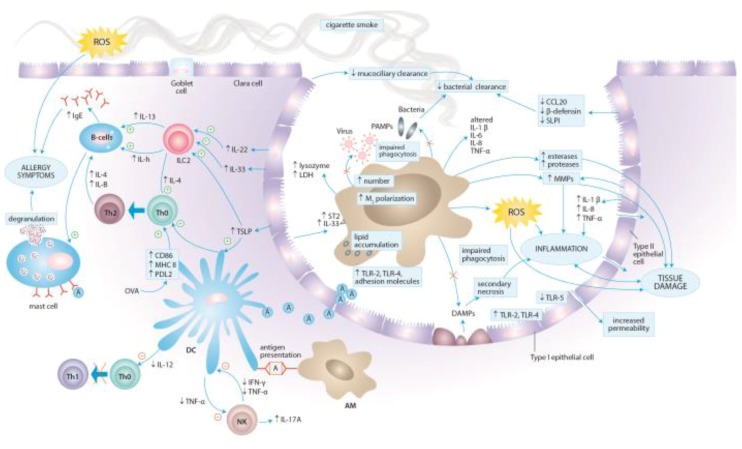
Local immune responses to cigarette smoke in the lung tissue.

The lung is directly exposed to environmental antigens including pathogens, allergens and toxins, such as tobacco smoke. A wide range of host defense mechanisms involving both innate and adaptive immune responses has been developed to provide protection against noxious agents. The weight of evidence indicates that chronic exposure to tobacco smoke alters the immune and inflammatory processes in the lung causing changes in humoral and cell-mediated immune responses (Figure 3) [99]. The influence of cigarette smoke on the immune system is, however, diverse and of dual nature—pro-inflammatory and immunosuppressive. Owing to the differences in smoking pattern (how the cigarette is smoked, number of puffs, puff volume, puff duration, etc.) as well as the age, sex, origin and socioeconomic status, the effects of tobacco smoke on the immune system can vary between the smokers and passively exposed individuals. Our understanding of CSE-induced effects is further complicated by sometimes contradictory results of human studies and experimental set-ups, probably due to the chemical heterogeneity of cigarette smoke, individual genetic susceptibility, and the variability in experimental methodologies (e.g., time, frequency and mode of exposure). Further to this, it seems that the amount of inhaled total particulate matter (TPM) has a great influence on the extension and nature of response to cigarette smoke. Indeed, Dvorkin-Gheva et al. has recently demonstrated that cigarette smoke-induced inflammation is a function of TPM with low TPM concentrations activating xenobiotic and detoxification mechanisms and high TPM concentrations driving additional inflammatory response potentially triggering tissue damage [100]. Therefore it must be noted, that the majority of available and reviewed here reports are based on animal models and cell lines studies. It is worth to emphasize that, the effects of passive smoking in experimental set-ups cannot be directly translated into the consequences of environmental tobacco smoke exposure in children.

The pro-inflammatory properties of cigarette smoke are well documented [101,102,103]. Cigarette smoke promotes inflammation by inducing the production of pro-inflammatory cytokines, such a TNF-α, IL-1, IL-6, IL-8 and granulocyte-macrophage colony-stimulating factor (GM-CSF), and increasing the accumulation of immune cells in the airway [104,105]. On the other hand, the results of many in vitro studies provide evidence for its immunosuppressive properties [2,106,107]. Specifically, the inhibitory effects of cigarette smoke have been related, among others, to nicotine. Nicotine was shown to decrease IL-6, IL-8, and IL-10 production [108]. One of the potential nicotine-induced immunosuppressive pathways is associated with the activation of its α7 nicotinic acetylcholine receptor on macrophages, T cells and B cells. Importantly, this activation was shown to suppress Th1 and Th17 responses with reciprocal shift towards the Th2 lineage [107]. This is further complicated by compounds demonstrating both pro-inflammatory and immunosuppressive properties, such as acrolein - another major component of tobacco smoke. While inhalation of acrolein promotes airway hypersensitivity responses, it may stimuli neutrophil accumulation in the airway, thereby contributing to immune tolerance [109].

### 4.1. Effects of Cigarette Smoke Exposure on Innate Immunity

The innate defense mechanisms of the airways and lungs encompass structural components, cough reflex, mucociliary clearance, epithelial barrier, humoral factors (surfactant proteins, complement proteins, antimicrobial peptides), and cells that elicit immune responses (epithelial cells, macrophages, monocytes, dendritic cells, neutrophils, natural killer cells, and mast cells). On the one hand, studies by Botelho et al. and D’Hulst et al. demonstrated that innate immune mechanisms are sufficient for driving cigarette smoke-induced inflammation in the airway [12,110]. There is several fold increase in the number of neutrophils, macrophages and dendritic cells in the airways of smokers and smoke-exposed animals in comparison to controls [14,111,112]. This influx of inflammatory cells leads to the aggravation of inflammatory processes, release of oxygen species, cytokines and chemokines, and activation of proteases. On the other hand, cigarette smoke suppresses local innate host defense in the airway as exemplified by a decrease in surfactant proteins SP-A and SP-D production. While SP-A and SP-D stimuli the phagocytosis of certain microorganisms by leukocytes, down-regulation of their synthesis clearly contributes to smoke-induced immunosuppression [113].

#### 4.1.1. Epithelial Cells

The respiratory epithelium is the first line of defense against environmental insults, in particular pathogens, inspired noxious particles, and allergens. However, airway epithelium does not merely serve as a physical barrier impeding the penetrance of potentially injurious materials but plays a pivotal role in the regulation of fluid balance, the metabolism and clearance of inhaled agents, and the regulation of immunological and inflammatory responses by secreting inflammatory mediators and recruiting immune cells [114]. In regard to allergic sensitization to inhaled antigens, airway epithelium is capable of recognizing allergens through expression of pattern recognition receptors (PRRs) and mounting innate immune responses [115].

Airway epithelium is composed of a variety of specialized epithelial cell types such as ciliated, mucous, goblet, Clara, and basal cells in the bronchial epithelium, and Type I and Type II cells in the alveolar epithelium [116]. To form a relatively impermeable barrier these cells are joined by tight and adherent junctions which form the apical junctional complex (AJC). Physical barrier is also maintained by mucociliary barrier comprising cilia, a periciliary fluid layer (sol), and a mucus layer (gel). Mucociliary clearance is provided by organized ciliary movements which remove pollutants and inhaled particulate material from the distal airway toward the pharynx [117]. CSE distorts the structure and function of the ciliary epithelium in a number of mechanisms. The results of in vitro and animal model studies show increased airway resistance and thickening of airway walls, increased number of mucous secreting goblet cells and mast cells. A concurrent decrease in number of Clara and ciliary cells leads to suppressed secretion of numerous anti-inflammatory, immunomodulatory, and antibacterial molecules that are vital to the host defense against pathogens [118,119,120,121,122,123,124,125].

Other smoking-induced changes include increased permeability of the respiratory epithelium and impaired mucociliary clearance resulting from mucous overproduction, decreased ciliogenesis, cilia shortening and decreased ciliary beat frequency [117,118,120,126,127,128,129]. The increase in epithelial permeability is associated with alterations in cytoskeletal and AJC structure and function as well as with changes in ion transport and lower trans-epithelial resistance [121,126,127,128,129,130]. In addition, genes involved in tight junctions formation, intraflagellar transport gene and cilia-related genes are markedly down-regulated in the airway epithelium of healthy smokers when compared with nonsmokers [131]. Smoking was also associated with higher MUC5AC-core gene expression [132]. MUC5AC is one of the predominant forms of mucin in the human airway that may represent an acute response to environmental insults [116]. Increased expression of MUC5AC in smokers may lead to mucus hypersecretion and subsequent mucociliary clearance impairment. Recently, cigarette smoke has been shown to increase TLR3-stimulated MUC5AC production in airway epithelial cells, mainly via extracellular signal-regulated kinases (ERK)-signaling [133]. The effect was partially attributed to the oxidative stress.

Cigarette smoking alters the inflammatory responses generated by the respiratory epithelial cells by modulating the production of a number of potent pro-inflammatory cytokines and chemokines with consecutive recruitment of macrophages and neutrophils and further damage to the lung tissue. This pro-inflammatory response of epithelial cells to CSE is achieved by altering a variety of signaling pathways involved in cellular activation, primarily protein kinase C (PKC), mitogen-actived protein kinase (MAPK), NFκB and activatory protein-1 (AP-1) pathways [102,134,135,136]. For example, exposure to cigarette smoke of human bronchial epithelium cell lines augmented the release of neutrophil chemoattractant IL-8, IL-1β, monocyte chemoattractant protein 1(MCP-1), TNFα, soluble intercellular adhesion molecule 1 (sICAM-1), and GM-CSF [101,102,104,137,138]. While IL-8 is one of the most potent neutrophil chemoattractants, IL-1β induces the activation of macrophages and the release of neutrophils from bone marrow via promoting GM-CSF production, and thus may play a role in enhancing oxidative burst and sustaining tissue inflammation in the airways [139]. Destruction of lung tissue is further facilitated by the accumulation of neutrophils, monocytes and NK cells in the airway during smoke-induced inflammation and increased expression of MMPs [140,141]. Although cigarette smoke is a potent inducer of neutrophilic inflammation, it was also shown to induce in the airway epithelial cells the synthesis of thymic stromal lymphopoetin (TSLP), a known activator of dendritic cells promoting Th2 polarization [142]. Airway epithelial cells are the main source of TSLP, IL-25, and IL-33, which govern upstream of the canonical Th2 cytokines: IL-4, IL-5, and IL-13 and thus induce a THh2-type immune response [143]. Therefore, increased production of TSLP is a potential mechanism by which cigarette smoke may prime allergic inflammation in the airway [142,144].

Epidemiological data clearly show that cigarette smoke exposure facilitates upper and lower respiratory tract infections. The identification of exclusive microbial molecules, pathogen-associated molecular patterns (PAMPs), is mediated through PRRs expressed on the surfaces of epithelial cells, macrophages and dendritic cells. PPRs families include Toll-like receptors (TLRs), purinergic receptors (e.g., P2X and P2Y), cytosolic nucleotide-binding oligomerization domain (NOD)-like receptors (NLRs), and receptor for advanced glycation end products (RAGE). While PRRs are required for elaboration of inflammatory response to invading pathogens, they are also activated by endogenous molecules released from injured or dying cells called damage-associated molecular patterns (DAMPs), such as double-stranded DNA, high-mobility group box 1, heat shock protein 70, mitochondrial DNA, and ATP. Cigarette smoke is a known inducer of necrotic and apoptotic cell death with subsequent DAMPs release [145]. Recently, cigarette smoke-induced epithelial necroptosis and DAMPs release have been shown to augment the release of CXCL8 and IL-6, in a myeloid differentiation primary response gene 88-dependent fashion [146]. It seems plausible, that cigarette smoke induces airway inflammation by direct oxidative damage to the epithelium as well as by activation of a variety of PRRs through DAMPs released following cell injury. Indeed, even short exposure to cigarette smoke is sufficient to increase the secretion of pro-inflammatory cytokines and the expression of several TLRs (e.g., TLR-2 and TLR-4) in epithelial cells [105,137,139,147,148]. Mortaz et al. demonstrated that cigarette smoke-induced release of CXCL8 is mediated through TLR4, TLR9 and inflammasome activation, and that P2X7 receptors and reactive oxygen species are involved [149]. In addition, the activation of purinergic receptor P2X7 plays a central role in NLRP3 inflammasome and caspase 1 activation, which facilitate the release of IL-1β and IL-18 [150,151]. Despite these results, much ambiguity remains surrounding the impact of cigarette smoke on the expression of TLRs. In particular, TLR-5 has been shown to be downregulated in the airways of smokers, which may account for the smoking-related susceptibility to airway infection by flagellated bacteria [152].

On the other hand, CSE exerts immunosuppressive effects on antimicrobial defense mechanisms of the airway epithelium. The stimulation of smoke-exposed epithelial cells with PAMPs, such as lipopolysaccharide (LPS) or dsRNA as well as exposure to human rhinovirus, nontypeable *Haemophilus influenzae* (NTHI), *Staphylococcus aureus* and *Moraxella catarrhalis* attenuated the in vitro production of potent pro-inflammatory mediators, in particular pathogen-induced neutrophil-mobilizing cytokines [136,153,154,155,156]. GM-CSF and IL-8 protein release from epithelial cells in response to LPS, a component of the outer membrane of Gram-negative bacteria, has been decreased following cigarette smoke exposure [136]. Similarly, stimulation of epithelial cells with poly (I:C), a viral double-strand RNA (dsRNA) mimic, led to a dose-dependent decrease in initiating an antiviral response and interferon production [153]. Additionally, the ability of epithelial cells to synthetize antimicrobial peptides, such as CCL20, β-defensin 1 and 2, and SLPI is suppressed in the presence of cigarette smoke [56,157]. This decrease in innate immune responses contributes to impaired defense against bacterial and viral infections, delayed pathogen clearance and chronic colonization of the lower airways by pathogens [158,159]. Recently, an enhanced adhesion of bacteria to the airway epithelium was demonstrated for *Streptococcus pneumoniae* [160]. Furthermore, blunted antimicrobial response seems to be associated with CS-induced modulation of cytoskeleton organization and inflammatory cell profiles during infections, which may contribute to further destruction of the lung tissue [161,162]. However, the exact mechanisms underlying cigarette smoke-induced impairment of defense against bacterial and viral agents are not completely understood and further research is warranted.

In aggregate, epithelial cells act as a first line defense against the deleterious effects of cigarette fume. Exposure to cigarette smoke leads to increased permeability of the respiratory epithelium, mucous overproduction, impaired mucociliary clearance, enhanced release of pro-inflammatory cytokines and chemokines with consecutive recruitment of macrophages and neutrophils, as well as altered pathogen sensing and clearance.

#### 4.1.2. Alveolar Macrophages

Alveolar macrophages (AMs) represent the most abundant immune cell type in the healthy airspaces. They are the most prominent phagocytes and antigen-presenting cells in the lung, and together with epithelial cells constitute the first line defense against infections and noxious agents. Apart from immune surveillance, responses to infections and microbial clearance, their functions comprise removal of cellular debris, maintenance of pulmonary tissue homeostasis and orchestrating the resolution of inflammation.

Numerous studies to date have proved that the exposure to cigarette smoke increases the number of alveolar macrophages in the airway by several fold and induces changes in their morphology and phenotype [163,164]. Distinctive morphologic changes of AMs caused by cigarette smoke include an increase in cell size as well as an increase in the number of Golgi vesicles, endoplasmic reticulum, and residual bodies, which contain distinctive fiber-like inclusions [165]. An increase in cell size can be partially attributed to intracellular lipid accumulation. Shortly after exposure to cigarette smoke AMs accumulate lipid droplets, presumably due to surfactant lipid oxidation. This leads to augmented IL-1β and GM-CSF production and initiates lung inflammation [166,167].

Cigarette smoke alters the expression of adhesion molecules on the surface of AMs obtained from smokers [164,168]. AMs in induced sputum of smokers expressed CD11b, CD14, CD54 and CD71 to a greater extent than AMs from nonsmokers, and the expression of CD11b and CD14 was associated with severe airflow limitation [164]. These alternations may possibly affect the metabolic activity, inter-cellular communication, adhesion, proliferation and maturation of alveolar macrophages [169]. Indeed, alveolar macrophages of smokers display higher resting metabolism, increased lysozyme secretion and lactate dehydrogenase, esterase and protease activity when compared with nonsmokers [165,170,171].

Macrophages show a significant phenotypic plasticity allowing them to adapt to the environment to which they are exposed. According to their activation status, macrophages have been broadly classified as either classically activated M1 macrophages or alternatively activated M2 macrophages, in parallel with T helper cells polarization [172]. TLR signaling and cytokines secreted by Th1 lymphocytes, such as interferon-γ (IFN-γ), induce M1 phenotype. M1 macrophages exhibit enhanced antimicrobial properties, release pro-inflammatory cytokines such as TNF-α, IL-6 and IL-12, and thus promote a Th1 environment [173]. The switch towards M2 polarization is generally induced by IL-4 and IL-13 [174]. M2 macrophages display an anti-inflammatory profile and produce anti-inflammatory cytokines such as IL-10 and transforming growth factor-β (TGF-β). M2 macrophages are involved in the encapsulation and destruction of parasites, immunoregulation, matrix deposition and tissue remodeling. It has been shown that the exposure to cigarette smoke induces a unique macrophage polarization pattern marked by a suppression of M1 and an induction of M2-related genes signatures [175,176,177]. The impact of cigarette smoke on macrophage polarization is multifaceted and not limited to changes in the lung cytokine milieu. The secretion of protease serine member S31 (Prss31) from bone marrow-derived mast cells was associated with macrophage infiltration and increased M2 polarization [178]. Moreover, cigarette smoke can change not only the phenotype of macrophages but can also direct blood and bone marrow monocyte polarization [179,180]. This skewed macrophage polarization finds its reflection in recently demonstrated heterogeneous airway deposition of macrophages in smokers and COPD patients [181]. A predominance of pro-inflammatory M1 cells was found in the small airways, whereas M2 macrophages dominated in the luminal areas. Both healthy smokers and COPD patients presented increased levels of M2 phenotype cytokines in bronchoalveolar lavage fluid (BAL) corresponding to M2 profile of luminal macrophages. The impact of cigarette smoke on macrophage phenotype polarization has implications well beyond COPD. Indeed, M1 and M2 macrophages contribute to the pathogenesis of asthma [173]. M1 macrophages release IL-23 and IL-1β that promote Th1 and Th17 responses implicated in airway neutrophilia and acute airway hyper-responsiveness. IL-14 and IL-13, key asthma cytokines, induce macrophage polarization towards M2 profile, which further promotes Th2 environment and airway remodeling. In a mouse model of house dust mite (HDM)-induced asthma, macrophage phenotypes reflected the changes in the severity of allergic airway inflammation [182]. Higher numbers of M1 macrophages were observed in mice with less severe asthma while increased numbers of M2 macrophages after HDM exposure correlated with higher eosinophil count. Apparently M1 macrophages play a dual role in asthma, preventing allergic sensitization on the one hand, but contributing to the development of severe phenotype in already established disease on the other.

Alveolar macrophages are potent inducers of inflammation and tissue degradation. Lung inflammation in smokers is perpetuated by protease/antiprotease imbalance and direct and indirect cell damage associated with the release of activated O_2_ intermediates. Smoking induces the production and activity of MMPs responsible for extracellular matrix degradation, and alters the production of their biological inhibitors, tissue inhibitors of MMPs (TIMPs) released by alveolar macrophages. Levels of MMP-9 and MMP-12 were shown to be increased in BAL samples from smokers and both MMP-9 and MMP-12 were shown to account for most of the elastase activity driven by alveolar macrophages [183,184,185,186]. In aggregate, the imbalance between MMPs and TIMPs leads to insufficient lung tissue repair and contributes to the pathogenesis of emphysema in smokers. Furthermore, AMs of smokers display increased release of oxidants such as superoxide anion and H_2_O_2_ than macrophages of nonsmokers [187]. For example, the smokers’ macrophages are capable of oxidizing the active site of alpha 1-antitrypsin, the major anti-neutrophil elastase of the human lower airway, which leads to its inactivation and contributes to further lung injury [188].

Although the number of alveolar macrophages is increased in BAL of smokers, a vast body of evidence suggests that these cells are functionally impaired [189,190,191,192,193,194]. Specifically, the ability to release pro-inflammatory cytokines (e.g., Il-1, IL-5, Il-6, IL-8, IL-12, TNF-α, IP-10, MCP-1, MIP-1α, and VEGF) seems to be substantially reduced following CSE and oxidative stress appears to be implied [195,196]. This explains, at least in part, why AMs from smokers elaborate a blunted inflammatory response after LPS stimulation, which results in increased susceptibility to infections [193,197,198,199]. Indeed, the ability of alveolar macrophages to phagocytose bacteria and apoptotic cells is substantially compromised by cigarette smoke [159,197,200,201,202,203]. Reduced rate of bacterial clearance was demonstrated for such important airway pathogens as *H. influenzae*, *L. monocytogenes*, *L. pneumophila*, *P. aeruginosa*, *S. pneumoniae* and *C. albicans* [159,197,200,201,202]. The mechanisms underlying this well-recognized suppression of phagocytosis encompass smoke-induced alternations in the ability to sense PAMPs and to kill bacteria. In particular, a disturbed expression of PRRs on alveolar macrophages may affect the recognition of bacterial agents and modify subsequent intracellular signaling and downstream effector mechanisms. Recently, cigarette smoke was shown to reduce the abundance of NLRP3 protein, a nucleotide-binding oligomerization (NOD)-like receptor (NLPR), through facilitating its ubiquitination [204]. In parallel, CSE was associated with an impairment of TLR-2 and TLR-4-associated signaling pathway activation in alveolar macrophages in response to agonists (e.g., LPS) and *Aspergillus* infection [205,206,207]. In short, TLR2 plays a role in recognition of lipoproteins and lipopeptides from Gram-positive bacteria, Mycoplasma, and mycobacteria, whereas TLR-4 is a key receptor for LPS from Gram-negative bacteria [208,209]. However, other results indicate, that cigarette smoke does not simply suppress AMs’ immune responses but rather skews their inflammatory profile [210]. Alveolar macrophages from smoke-exposed mice challenged with nontypeable *Haemophilus influenzae* elicited increased levels of inflammatory mediators, such as MCP-1, MCP-3, MCP-5, IP-10, and MIP-1γ, whereas the expression of pro-inflammatory cytokines, namely TNF-α, IL-1β, IL-6, was decreased in comparison with bacteria-challenged control mice. Of note, this altered inflammatory profile was associated with the exacerbation of the inflammatory response, which was neutrophilic in nature. Increased CXCL8 levels and neutrophil counts are typically found in the airway of COPD patients with bacterial colonization. In line, suppressing effect of CSE on pro-inflammatory cytokines expression does not apply for CXCL8, a potent neutrophil chemoattractant [199].

Due to impaired pathogen recognition and altered immune response, AMs from smokers exhibit reduced phagocytic properties. The weight of evidence indicates that CSE impairs bacterial clearance and enhances bacteria survival in the airway [159,202,211]. Exposure to cigarette smoke was associated with decreased bactericidal or bacteriostatic properties, suppressed fusion of phagolysosome and autophagy impairment probably related to oxidative stress [200,212,213]. Furthermore, cigarette smoke was shown to reduce major histocompatibility class I (MHC I) antigen presentation by suppressing the activity of immunoproteasome [214]. Since MHC I-mediated antigen presentation to CD8+ T cells is crucial for mounting immune response against virus-infected cells, cigarette smoke may dampen antiviral immune responses. This is in line with diminished MHC I levels on alveolar macrophages observed in smokers with COPD [215].

Apart from ineffective bacterial clearance, impaired phagocytic properties of alveolar macrophages account for deffective phagocytosis of apoptotic bronchial epithelial cells, a process termed ‘efferocytosis’. Defective efferocytosis facilitates leakage of apoptotic cell contents into the surrounding tissue exposing neighboring cells to noxious intracellular components, such as enzymes (e.g., proteases and caspases) and oxidants. Accumulated apoptotic cells may undergo secondary necrosis contributing to a perpetuation of inflammation and resulting in tissue damage persistent even after smoking cessation [145]. Disturbed efferocytosis with abnormal accumulation of apoptotic epithelial cells in the airway lumen has been conclusively documented in COPD patients [216]. Cigarette smoke was shown to reduce the expression of some recognition molecules on the surface of alveolar macrophages, namely CD31, CD91, CD44, and CD71, which are necessary for epithelial/macrophage crosstalk and effective clearance of apoptotic cells and tissue debris [217]. Impaired efferocytosis by alveolar macrophages appears to be an important contributor to the exacerbated cellular inflammation not only in COPD, but also in asthma, bronchiolitis obliterans, protracted bacterial bronchitis and bronchiectasis in children [216,218,219,220,221]. Recently, considerable emphasis has been placed on the role of sphingolipid metabolites in cigarette induced-efferocytosis impairment with sphingosine 1-phosphate (S1P) signaling garnering especially great attention [222,223,224,225]. S1P downstream signaling pathways participate in innate and adaptive immune responses, in particular in leukocyte trafficking and differentiation. Recently found disparity in the expression of Spinster 2 (Spns2), a plasma membrane transporter of S1P, between AMs and epithelial cells suggests, that cigarette smoke-induced impairment of AMs’ phagocytic properties may be a consequence of disturbed crosstalk between these two cell types [223]. These observations are of interest as S1P signaling was shown to be involved in airway inflammation and hypersensitivity as well as delayed-type contact hypersensitivity [226]. In ovalbumin (OVA)-induced allergic asthma model, Spns2-knockout mice exhibited decreased count of eosinophils and lymphocytes as well as increased macrophage numbers in BAL fluid, elaborated blunted Th2-type response with significantly decreased levels of IL-4, IL-13 and IL-5, and diminished antigen-specific antibody production when compared with wild type littermates. These results indicate, that Spns2 is implicated in both the induction of Th2-type inflammatory response in the airway, a hallmark of allergic asthma, and the regulation of Th1-driven inflammation in skin diseases [226].

Taken together, the net effect of cigarette smoke on alveolar macrophages is a significant increase in AM count with concurrent substantial impairment of their function. Decreased phagocytic ability of AMs contributes to augmentation of inflammation, increased susceptibility to respiratory infections and tissue damage.

#### 4.1.3. Dendritic Cells

Dendritic cells are the most important antigen-presenting cells uniquely positioned at the interface between the innate and adaptive part of the immune system [227,228]. Human DCs can be divided into convetional DCs or “myeloid” DCs (mDCs) and plasmocytoid DCs (pDCs). DCs govern differentiation and activation of antigen-specific T-cells in response to pathogens. This is mediated by antigen presentation, co-stimulatory molecule expression, and immune-stimulatory cytokine release [229]. Depending on the character of these signals, DCs may determine T-cell response polarization.

Active smoking substantially impacts the number, distribution, and differentiation of DCs and Langerhans Cells (LCs) in human alveolar parenchyma [230]. Cigarette smoking has the capacity to lower the number of bronchial mucosal DCs in COPD and asthma patients [231,232]. In contrast, short smoke exposure was shown to increase CD11b+ DCs count in bronchoalveolar lavage fluid, which was associated with sensitization and asthma development [233].

Several separate lines of evidence suggest that cigarette smoke exposure impairs the maturation and function of DCs. Cigarette smoke-exposed DCs displayed diminished T-cell-stimulatory capacity [234]. In asthmatic rats, the expression of myeloid differentiation factor 88(MyD88), IL-10 and IL-12 was decreased in marrow DCs as a result of cigarette smoke exposure [235]. In parallel, DC maturation within the lymph nodes was impaired by cigarette smoke, as demonstrated by reduced cell surface expression of MHC II and the costimulatory molecules CD80 and CD86. These DCs had a diminished capacity to induce IL-2 production by T-cells. Of note, DC-induced T-cell function impairment may lead to the exacerbations of COPD, diminished infection response, and inhibited tumour surveillance [236]. However, in another murine model cigarette smoke extract was shown to induce neutrophil extracellular traps, which in turn were capable of driving plasmacytoid DCs (pDCs) maturation and activation [237].

CSE has a definite impact on DCs response to infections. While both subtypes of DCs express PRRs allowing effective pathogen sensing, only pDCs express TLR-9 which facilitates the recognition of viral double-stranded DNA. Therefore, pDCs constitute an important driver of innate antiviral immunity. CSE was shown to augment the production of IL-8, a potent neutrophil chemoattractant, with simultaneous suppression of the pro-inflammatory cytokine release (TNF-α and IL-6) after TLR-9 induction. Importantly, CSE attenuated IFN-α, a key antiviral protein, production through the suppression of PI3K/Akt signaling pathway. Taken together, these data indicate that CSE has the potential to lessen anti-viral immunity with concurrent induction of neutrophilic inflammation [149]. Accordingly, CSE downregulated the expression of TLR-7 and the activation of interferon regulatory factor (IRF)-7 in RSV-infected pDCs. RSV-induced release of INF-α, Il-1β, Il-10 and CXCL10 was inhibited in pDCs further demonstrating that the key functions of pDCs upon viral infection are impaired by CSE [238]. In parallel, cigarette smoke alters the ability of DCs to promote anti-bacterial response. Nicotine was shown to reduce both endocytic and phagocytic abilities of immature DCs. The production of cytokines such as Il-10, Il-12, Il-1β and TNF-α in response to bacterial products was impaired [239]. In particular, the suppression of IL-12 production, a potent inducer of Th1 responses, may potentially contribute to blunted host defense mechanisms against infections mediated by DCs exposed to nicotine. Accordingly, CSE was observed to significantly reduce S. pneumoniae-induced monocyte derived DC maturation and to suppress DC capacity to activate antigen specific T-cell response. Specifically, a decrease in pro-Th1 and -Th17 response accompanied by sustained CXCL8 secretion accounted for impaired anti-bacterial defense mechanism and aggravated neutrophil airway deposition [234]. Data regarding cigarette smoke impact on cytokine production in DCs are, however, conflicting. While suppressed production of inflammatory mediators (IL-12 and IL-23) was attributed to reactive oxygen species or nicotine, stimulation with LPS or CD40 ligand resulted in up-regulation of certain inflammatory mediators (prostaglandin-E2, IL-8, and IL-10) [240,241,242].

There is much evidence suggesting cigarette smoke contributes to inflammation and allergic response mediated by DCs. In murine models, short smoke exposure has been shown to amplify dendritic cell-mediated transport of house dust mite allergens to the intrathoracic lymph nodes and to generate a local T-helper cell type 2 response. This was accomplished by selective up-regulation of C-C chemokine receptor 7 (CCR7) surface expression on airway DCs promoted by cigarette smoke. The CCR7 protein is crucial to the migration of DCs to lymphatic nodes, which is a key step in the antigen-presenting function of airway DCs. The authors conclude that the concomitant inhalation of aerosolized ovalbumin (OVA) and cigarette smoke induces Th2-type airway inflammation, which was not observed after exposure to either agent alone. Cigarette smoke was also shown to induce up-regulation of MHC class II, CD86 (B7-2), PDL2 and down-regulation of Inducible T-Cell Costimulator Ligand (ICOSL) on airway DCs. CD86 is a costimulatory molecule involved in the priming of Th2 responses and the subsequent development of allergic airway inflammation. Thus, cigarette smoke may activate pulmonary DCs in a way that promotes allergic sensitization against co-inhaled molecules. Such skewing of immune response appears to be TLR-independent and is attributed to the presence of immunological adjuvants in the smoke fume [243]. Subsequent study, however, using a murine model of allergic pulmonary inflammation, revealed that cigarette smoke effects on DCs do not simply promote allergic airway inflammation, but rather alter the Th1/Th2 balance. Mice previously sensitized and challenged with OVA demonstrated a decreased number of eosinophils and suppressed IL-4 and IL-13 production following cigarette smoke exposure. Cigarette smoke exposure associated with OVA sensitization reduced the number of pDCs and their activation by suppressing the expression of CD86, PDL2 and ICOSL, which was sufficient to decrease regulatory T cells recruitment and IL-10 and TGF-β production. Additionally, cigarette smoke increased the recruitment of CD8α^+^ DCs into lymph nodes, which may account for an increase in number and activation of CD8+ T cells in the lungs [244].

In conclusion, cigarette smoke may alter the immune profile of DCs in a variety of ways, some of which are contradictory. One possible explanation for the apparent paradox was proposed by Nouri-Shirazi and Guinet who stated that DCs developed in a nicotinic environment fail to support the terminal development of effector memory Th1 cells due to their differential expression of costimulatory molecules CD86 and CD80 and lack of IL-12 production [245]. It is plausible, that DCs can adopt Th-1 promoting function, which is necessary to fight infections, only when the balance of environmental signals strongly favors Th1 immunity, and promote Th2 response in a Th2-biased environment, which primes the development and exacerbation of asthma [246].

#### 4.1.4. Natural Killer Cells

Natural Killer (NK) cells are large granular lymphocytes similar to cytotoxic lymphocytes able to secrete perforin, granzymes, TNF-α and IFN-γ but unable to rearrange T-cell receptor or immunoglobulin genes [247]. NK cells are responsible for defense against microbial agents and tumour surveillance. This is mediated by Ca^2+^-dependent granule exocytosis, cytotoxic proteins (perforin and granzymes) release from intracytoplasmic granules, constitutive or induced upon interaction with target cells FasL expression, Ca^2+^-independent Fas (CD95/Apo 1)-mediated apoptosis induction, and membrane-bound or secreted cytokines (e.g., TNF-α) production [248].

CSE has been reported to both suppress and stimulate the activity of NK cells. NK cell activity in peripheral blood was reduced in smokers compared with non-smokers. These alterations appear to be reversible, since a recovery period of six weeks after smoking cessation brought the cytotoxic activity of NK cells back to the level of never-smokers [249]. NK cells from long-term smokers display a decreased intracellular IL-16 concentration. This depletion of the CD4+-recruiting cytokine strongly suggests that long-term smoking may impact immune responses at the systemic level, and that NK cells are involved [250]. There is ample evidence showing direct negative effect of cigarette smoke on NK cell cytolytic capacity, as well as on their ability to produce inflammatory cytokines in response to microbial agents. Mian et al. demonstrated that cigarette smoke significantly suppresses the induction of IL-15 by polyinosinic:polycytidylic acid (poly I:C) in human peripheral blood mononuclear cells (PBMCs). This decrease in IL-15 production, which was linked with attenuated signal transducer and activator of transcription (STAT) 3 and STAT5 phosphorylation, compromises NK cell cytolytic potential [251]. NK cell TNF- α and IFN-γ production was also shown to be impaired after treatment with tobacco product preparations and stimulation with poly I:C and LPS [252]. Cigarette smoke has been found to inhibit IFN-γ production in vitro and ex vivo by poly I:C induced NK cells. TNF-α production after stimulation with poly I:C was also decreased in smokers compared with non-smokers, as was perforin expression and NK cell cytotoxic activity [253].

These data notwithstanding, cigarette smoke may also exert a stimulatory effect on NK cells. Stolberg et al. show that acute cigarette smoke exposure elicits NK cell activation. In particular, cigarette smoke exposure caused increased accumulation of primed/activated CD69(+) NK cells in parenchymal and mucosal locations in the airway. The priming and activation of NK cells is believed to result from crosstalk between NK and sentinel cells, such as DCs, and CCR4 appears to be a possible promoter of NK/DC interaction [254]. Furthermore, CSE up-regulates epithelial-derived IL-33 level with reciprocal increase in IL-33 receptor ST2 expression on macrophages and NK cells. This can significantly contribute to the amplification of type I proinflammatory responses within the lung during infection [255]. In line, analysis of human data showed that acute smoking was associated with systemic activation of NK cells. The activation of pulmonary NK cells was dependent on COPD coincidence, regardless of current smoking status [256].

In summary, the impact of smoking on cytokine production and cytolytic activity of NK cells is ambiguous, with evidence pointing at both pro-inflammatory and anti-inflammatory effects. The overall result is likely dependent on the existing comorbidities and general state of the patient.

#### 4.1.5. Neutrophils

Neutrophils play a pivotal role in the pathogenesis of COPD. However, considerable emphasis has been recently placed on neutrophilic inflammation in asthma [257]. According to epidemiologic and experimental studies, cigarette smoke is a potent inducer of neutrophilic inflammation. In short, CSE leads to necrosis of bronchial epithelial cells with reciprocal DAMPs release and pro-inflammatory cytokine production, which, in turn, induce neutrophilic airway inflammation [146]. Higher numbers of neutrophils in BALF following CSE have been repeatedly demonstrated in humans and experimental models [146,258,259]. In patients with asthma, neutrophilic airway inflammation is frequently associated with severe course of the disease and poor response to glucocorticoid therapy. Asthmatic smokers have higher expression of IL-17A, IL-6 and IL-8, and neutrophil numbers in the bronchial mucosa when compared with non-smoking asthmatics [260]. Further to this, co-stimulation with CSE, smoke-induced IL-17A and aeroallergens further increases IL-6 and IL-8 production, indicating that smoke-induced neutrophilic inflammation in asthmatics may be self-sustaining in nature.

CSE was also shown to alter neutrophil activation and chemotaxis which may contribute to impaired immune responses observed in smokers. There is some debate as to whether CSE can trigger the formation of neutrophil extracellular traps (NETs), which are critical for antimicrobial host innate defence responses. Cigarette smoke exposure appeared able to trigger neutrophils to undergo NETosis. Moreover, CSE-induced NETs were shown capable of driving murine plasmacytoid dendritic cells maturation and activation with ensuing polarization of naive CD4+ T cells towards Th1 and Th17 responses [237]. A more recent study, however, demonstrated that the release of NETs was impaired in human neutrophils from the peripheral venous blood of heathy volunteers after short treatment with CSE or its components or metabolites. Additionally, these neutrophils exhibited decreased speed, velocity and directionality [261].

In conclusion, the majority of evidence seems to suggest that cigarette smoke exposure is associated with the induction of neutrophilic inflammation, the primary sign of which is an increase in neutrophil numbers. It may, however, alter the response to infectious agents, with studies showing both decreased and increased chemotaxis and activation after exposure to microbial products.

### 4.2. Effects of Cigarette Smoke Exposure on Adaptive Immunity

In response to pathogens, innate immune responses mount a more sophisticated, pathogen-specific adaptive immune response. It is now well appreciated that cigarette smoke has a profound impact on activity and function of adaptive immune cells, namely T helper cells (Th1, Th2, Th17), CD4+CD25+ regulatory T cells, CD8+ T cells, B cells and memory T and B lymphocytes.

#### 4.2.1. T Lymphocytes

T cells play a central role in cell-mediated adaptive immunity. Following antigen recognition innate immune cells activate and stimulate naïve T cells to differentiation with ensuing generation of predominantly effector T cells and, to a lesser extent, memory and regulatory T cells [262,263,264,265,266].

Cigarette smoke exposure leads to significant changes in certain T cell subtypes prevalence in blood and tissues. Active smokers were shown to have higher numbers of circulating CD3+ T, CD4+ T, and total lymphocytes than nonsmokers [267]. Consistently, numbers of memory T cells and class-switched memory B cells were significantly and positively correlated with smoking habits, indicating that lymphocytes are sensitive to cumulative effect of smoking [267,268]. While active smoking exerts positive effect on memory T-cell counts, this may not be true for passive smoking. In nonsmoking adolescents exposure to SHS was correlated with reduced numbers of circulating CD3+ and CD4+ memory T-cells with a reciprocal linear increase in the percentage of naïve CD4+CD45RA+ and CD3+CD45RA+ T-cell subsets [269].

Other studies demonstrated higher numbers of circulating Th17 cells and, although not consistently, increased percentages of CD8+ T-cells in smokers or COPD patients [270,271]. Vargas-Rojas et al. has shown that the percentage of Th17 cells in circulating T cell subsets from COPD patients was higher than Th17 levels in healthy population [272]. Furthermore, cigarette smoke exposed mice had higher numbers of IL-21+ Th17 and IL-21R+ CD8+ T cells in peripheral blood. The impact of CSE on the numbers of circulating CD8+ T-cells is, however, ambiguous. In contrast to previous study, Koch et al. showed that smokers with COPD have less circulating CD8+ T cells than smokers without COPD and nonsmokers, and that these cells have decreased chemotactic activity. However, the percentage of circulating cytotoxic effector CD8+ T-lymphocytes was increased in smokers and COPD patients compared with nonsmokers [271].

Studies assessing T cell composition in BAL fluid demonstrated that smokers have a decreased CD4+/CD8+ ratio when compared with nonsmokers [273]. This change in T cell subsets is caused by higher proportion of CD8+ T lymphocytes in BAL fluid which has been repetitively found in smokers and COPD patients [274,275]. Furthermore, smokers exhibited a higher Tc1/Tc2 CD8+ T cells subtypes ratio in BAL fluid associated with augmented IFN-γ production [275]. CD8+ T cells can be divided into two subtypes—Tc1 cells releasing IFN-γ but not IL-4, and Tc2 cells producing IL-4 but not IFN-γ. These data notwithstanding, chronic CSE was associated with elevated numbers of Th1 and Th17 cells in BAL fluid [276].

To sum up, cigarette smokers have an increased number of circulating CD3+ T and CD4+ T lymphocytes including Th17 cells, whereas the precise influence of cigarette smoke on CD8+ T cell number in peripheral blood remains unclear. CSE increases the numbers of CD8+, Th1, and Th17 cells in BAL fluid. Some discrepancies in results between the studies may, however, be found, yielding some uncertainty as to the precise mechanisms governing cellular recruitment to different airway environments.

##### Th1 and Th2 Cells

Type 1 T helper (Th1) and Th2 lymphocytes are two subsets of CD4+ T cells with reciprocal functions and patterns of cytokines. Th1 cells produce interferon-gamma, IL-2, and TNF-β, which activate macrophages and are responsible for cell-mediated immunity and phagocyte-dependent protective responses [277]. Th2 cells are characterized by IL-4, IL-5, IL-10, and IL-13 production, which are responsible for enhanced antibody synthesis, eosinophil activation, and inhibition of several macrophage functions, thus providing phagocyte-independent protective responses [277].

It has been shown that continuous cigarette smoke exposure alters the balance between Th1 and Th2 CD4+ T cells in the lung [278]. Chronic CSE was associated with elevated numbers of Th1 and Th17 cells in BAL fluid as well as with augmented Th2- mediated airway inflammation [16,276]. Increased percentages of Th1 and Th17 cells in smokers and COPD patients may be responsible for sustaining chronic pulmonary inflammation. On the other hand, CSE was shown to dampen Th1 type and to promote Th2 type immune responses [278]. While Th1 augmentation may increase the risk for the development of emphysema, Th1 to Th2 shift may favor development of allergic diseases such as asthma. In accordance, our group has previously demonstrated a Th2 driven immune responses in asthmatic children exposed to parental tobacco smoke [279]. Shaler et al. showed that continuous cigarette smoke exposure hinders anti-mycobacterial type 1 protective immunity in pulmonary tuberculosis murine models. Mechanistically, CSE considerably impaired lung deposition of antigen presenting cells and their production of IFN-γ, TNF-α, IL-12 and RANTES, thus inhibiting the recruitment of Th1 polarized cells to the lung. Concurrently, CSE enhanced Th2 CD4+IL-4+ responses in the lung, inclining that it may initiate specific changes in T-cell polarization upon entering the lung. Consistent with severely diminished Th1 cytokine production, continuous cigarette smoke exposure substantially inhibited the ability of lung mononuclear cells to produce nitric oxide, thereby diminishing the amount of bactericidal products in the lung and dampening anti-bacterial host defense responses. This is of great importance, as severely weakened Th1 immunity in the lung caused by tobacco smoke exposure predisposes to chronic bacterial colonization and infection, as exemplified by weakened mycobacterial control.

In support of the premise that CSE could bias local lung immune responses towards Th2 immunity come observations from experimental asthma models. Apparently, concurrent administration of cigarette smoke and ovalbumin induces the development of airway inflammation with predominant Th2-type immune responses and is further associated with delayed tolerance to inhalant antigen [16]. This shift towards Th2 immunity is primed by a plethora of mediators including lipocortin 1 and aforementioned TSLP [142]. Lipocortin 1 is a naturally occurring defense factor against inflammation with the capacity to regulate T helper responses. Bhalla et al. have observed that impaired synthesis and degradation of lipocortin 1 may bidirectionally influence immune responses in animals exposed to tobacco smoke either by augmenting T helper cell Th1 response or by shifting Th1 to Th2 response [280].

In aggregate, despite convincing evidence indicating that CSE enhances Th1 immune responses, which are of great importance in COPD pathogenesis and progression, data exist on CSE contribution to Th2 bias of lung immunity crucial for allergic sensitization and asthma.

##### Th17 Cells

T helper 17 cells (Th17) are a subset of pro-inflammatory T cells defined by their production of interleukin 17 (IL-17) [281]. IL-17A is a pro-inflammatory cytokine predominantly released from Th17 cells. It is a well-recognized regulator of cellular immunity since it has the capacity to stimulate the expression of secondary pro-inflammatory chemokines and growth factors in epithelial and mesenchymal cells and, thus, to mediate neutrophil recruitment and activation as well as neutrophil and macrophage accumulation in the lung tissue [282]. Of note, IL-17A has been implicated in the pathogenesis of asthma [283]. IL-17A expression is increased in blood eosinophils, sputum and bronchoalveolar lavage specimens of asthmatic patients in comparison with control subjects [284]. During allergen-induced airway inflammation IL-17A primes recruitment of alveolar macrophages and neutrophils and regulates airway hyper-reactivity to methacholine [283,285]. Moreover, IL-17 appears to play a crucial role in activating T-cells in allergen-specific immune responses, as IL-17-deficient mice showed attenuated T-dependent antibody production as well as contact, delayed-type, and airway hypersensitivity responses [285].

Evidence from human and experimental studies strongly suggests, that CSE increases the number of Th17 cells in lung tissue and peripheral blood [270,272,286]. Higher count of Th17 cells was associated with augmented expressions of IL-17 and IL-21 accounting for up-regulation of perforin and granzyme B production in increased number of CD8+ T-cells. In general, Th17 cells are negatively correlated with T regulatory cells and the signals that cause Th17s to differentiate inhibit Treg differentiation [281]. Therefore, these results may imply that cytotoxic function of CD8 + T cells can be regulated by Th17 cells [270]. In accordance, mice with COPD induced by constant tobacco smoke presented an increased level of Th17 subset followed by upregulation of Th17-series of cytokines (IL-6, IL-17A and IL-23) in both lung tissue and peripheral blood [286]. Moreover, CSE-induced Th17 responses are strongly implicated in the induction of several autoimmune diseases such as COPD, psoriasis and rheumatoid arthritis [287].

To conclude, findings from both animal and human studies highlight the fact that Th17 cells contribute to the intensification of smoking-induced inflammation and are associated with autoimmune responses.

##### Treg Cells

The regulatory T cells are a subpopulation of T cells that modulate the immune system, maintain tolerance to self-antigens, and prevent autoimmune disease. Tregs are immunosuppressive and generally suppress or downregulate induction and proliferation of effector T cells [288]. Many studies have demonstrated that tobacco smoke exposure leads to Treg imbalance. A study on phenotypic patterns of T-lymphocytes in COPD has shown a considerable downregulation of CD4+ CD25+ Treg cells in BAL fluid from patients with COPD compared with healthy smokers [289]. On the contrary, many studies have shown increased levels of Treg cells among COPD patients. Patients with diagnosed COPD who remained smokers had significantly increased level of CD4+CD25+ Tregs compared to healthy non-smokers [290]. Study by Hou et al. demonstrated unbalancing effects of tobacco smoke exposure on Treg cells [291]. Specifically, COPD patients had a decreased number of suppressive Tregs (CD25++ CD45RA+ resting Tregs and CD25+++ CD45RA− activated Tregs) but higher percentage of FrIII cells compared with non COPD smokers, which may imply that Treg imbalance (aTreg+rTreg vs. Fr III) has an impact on pathogenesis of COPD.

#### 4.2.2. B Lymphocytes

B cells play an important role as a component of the adaptive immune system. They are responsible for secreting antibodies and cytokines and participate in antigen presenting process. Compelling evidence from human and experimental studies suggests that cigarette smoking may be associated with the suppression of B-cell development, function and immunoglobulin production [292,293]. Additionally, cigarette smoke-induced alternations in B cell distribution and the underlying mechanisms have gained considerable attention recently. COPD patients were shown to have an increased number of B cells in the small airways [294]. A more recent study demonstrated lower (memory) B-cell counts with concurrent increase in Treg number in peripheral blood of COPD patients in comparison with healthy controls [292]. While the memory B-cell percentages in peripheral blood were significantly decreased, current smokers displayed higher percentages of class-switched memory B cells than non-smokers, regardless of the disease state. Since the process of class-switch recombination results from repeated antigen recognition, this finding suggests that cigarette smoke is potentially capable of generating neo-antigens derived from damaged lung tissue or smoke fume components in a chronic manner.

Consistent with the aforementioned findings regarding lower percentages of B cells in blood, cigarette smoke exposure can suppress B-cell differentiation process at a very early stage, as a significant down-regulation of pre-B and pro-B cells was identified in murine bone marrow [295,296].

Other deleterious effects of cigarette smoke exposure comprise the suppression of immunoglobulin production. Though the secretion of IgA, IgG and IgM appears to be down-regulated in peripheral blood and saliva of smokers, this suppressive effect does not affect IgE synthesis [297]. Indeed, smokers have increased levels of circulating IgE, which may potentially account for increased risk of atopy and asthma development [108,297]. A study conducted in 1983 provided evidence that the mean IgE level in ex-smokers was much lower than in current light smokers but was still higher than in nonsmokers [298].

Altogether, cigarette smoking increases airway deposition of B cells, decreases frequency of memory B-cells in peripheral blood, down-regulates secretion of IgA, IgG and IgM but augments the production of IgE, possibly contributing to allergic diseases. The negative impact of CSE on B-cells can be identified very early in differentiation process, as exemplified by the suppression of bone marrow pre-B cells and pro-B cells in mice.

## 5. Conclusions and Further Research

For the last decades, we have witnessed a substantial limitation of ETS exposure. We have better and better legal regulations, and the number of smokers is gradually decreasing (especially in Europe). However, a significant part of the population of the world is still exposed to the detrimental effects of tobacco smoking. Children are particularly vulnerable to harmful effects of cigarette fume. A chemically diverse mixture of pro-inflammatory, oxidative and carcinogenic factors found in tobacco smoke has a number of different, sometimes contradictory effects. In this review we demonstrated, in a comprehensive manner, the current state of knowledge on ETS effect upon immune function with a strong emphasis on the airway. Cigarette smoke alters a myriad of signaling pathways and immune responses, and some of them are implicated in the development of allergic diseases. We presented data on the molecular and cellular level from both animal models and clinical studies. It is worth noting that the mechanisms presented above may not only apply to ETS but also to other air pollutants, including traffic-related air pollution. Clinical and laboratory data on how environmental pollution contributes to the development of diseases of affluence, including allergy is accumulating. However this area definitely requires further research.
